# Understanding Motivation in Early Childhood: Disentangling the Links Among Curiosity, Mindset, and Goals

**DOI:** 10.3390/bs16010054

**Published:** 2025-12-29

**Authors:** Natalie Hutchins, Jamie Jirout

**Affiliations:** School of Education and Human Development, University of Virginia, Charlottesville, VA 22904, USA; jirout@virginia.edu

**Keywords:** curiosity, growth mindset, achievement goals

## Abstract

Children’s academic motivation declines with grade, beginning in early elementary school, so a better understanding of young children’s motivation is needed. Measuring motivational constructs in children is a necessary start to this goal with a focus on children’s curiosity, mindset, and achievement goal orientations—all shown to be consistently related to academic success across developmental periods. In 212 6–10-year olds, factor analyses showed separate factors for each of the expected constructs. Curiosity positively related to growth mindset instability—but not malleability—beliefs, and mastery goal orientations, and achievement goal orientations (performance, mastery) were positively associated, though they did not relate to growth mindset beliefs. Disentangling the observed associations that diverge from the prior literature can help to identify promising future directions for supporting children’s motivation and learning.

## 1. Introduction

One of the most consistent influences of academic performance and learning behaviors in elementary age students is motivation, but different motivational factors can influence different students in varying ways (Wigfield & Eccles, 2020). The current study focuses specifically on children’s thinking about why they learn, as these motivational constructs represent relatively stable academic dispositions (rather than task-specific states) that guide how children interpret and respond to learning challenges in academic contexts to support school achievement. Motivational constructs related to school achievement include curiosity (Shah et al., 2018; Gottfried et al., 2016), mindset (Claro et al., 2016; Dweck, 2006), and mastery orientation (Dweck, 1986; Nicholls, 1984). These constructs provide complementary aspects of motivation in education, including epistemic motivation with curiosity, which reflects a child’s intrinsic drive to seek information and reduce uncertainty, achievement motivation with goal orientations, which shape how students define success and respond to difficulty, and beliefs about ability with growth and fixed mindsets, which relate to children’s thinking about the malleability of intelligence. These constructs are likely to be related (Eccles & Wigfield, 2020) and malleable (Gilboy et al., 2015; Kpolovie et al., 2014; Nguyen et al., 2018), but are often studied independently with unclear associations, despite strong theoretical support for their likely bidirectional relations (Pekrun, 2024). Based on prior work showing the complexity of interacting motivational factors, understanding whether these constructs are empirically distinct and associated can inform methods for supporting or mitigating the decline observed in motivation beginning in early schooling (Scherrer & Preckel, 2019; Wang & Pomerantz, 2009), supporting motivational states (e.g., interest, self-efficacy) and contributing to persistence, exploration, and long-term engagement in school.

The constructs of interest are distinct in definition but overlap conceptually, all relating to how children make sense of learning opportunities and challenges. They represent tendencies to seek information, define success, and construe effort in particular ways, and each has been empirically linked to adaptive learning behaviors (Engel, 2011; Hidi & Renninger, 2006; Kaplan & Maehr, 2007; Miele & Molden, 2010). Curiosity is defined here as the desire to seek information in response to knowledge gaps, especially those stemming from uncertainty or ambiguity (J. J. Jirout, 2020). Importantly, curiosity seems to decline with schooling (Engel, 2011). Growth mindset is defined here as beliefs about intelligence as malleable with effort/behavior and with time; fixed mindset is the belief that ability and intelligence are static and inherent over time (Dweck, 2006). While curiosity draws learners into exploration, growth mindset beliefs shape how they interpret challenges and setbacks within that process. Having a growth mindset may be important for becoming curious (J. J. Jirout et al., 2018). Differing from the mindset about one’s ability, also important are the reasons for why learners are motivated, or the orientation of their achievement goals (Dweck, 1986; Nicholls, 1984). Mastery goals focus on enhancing understanding, with learning seen as inherently interesting and valuable, while performance goals center on others’ views of one’s competence (Dweck, 1986; Nicholls, 1984). Performance approach goals involve demonstrating competence; performance avoidance goals involve preventing the demonstration of incompetence (Dweck, 1986; Nicholls, 1984). Though different, these goals are not mutually exclusive—learners can hold different concurrent achievement goals (Harackiewicz et al., 2002).

Empirical work testing the associations among curiosity, growth mindset, and achievement goal orientations shows somewhat inconsistent results, and no study has included these constructs together in the same sample of children. One study showed that growth mindset beliefs and curiosity positively relate in Chinese sixth graders (Hong et al., 2017); another found that curiosity was uncorrelated with performance approach success and slightly negatively correlated with performance avoidance failure in adults (Litman, 2008). Students with growth mindsets generally view effort positively and pursue mastery goals to develop their competence (e.g., Blackwell et al., 2007). Specifically, within a meta-analytic review across age groups (range = 5–42 years old), growth mindset beliefs positively relate to having mastery goals and negatively relate to having performance approach and avoidance goals, with weaker associations when studying academic domains, and individual studies sometimes show no association or even negative associations (Burnette et al., 2013). However, mindset beliefs and goal orientations may interact. For example, students with growth mindsets can coordinate mastery and performance goals simultaneously, while those with fixed mindsets struggle to sustain mastery goals (Stone, 1999; Dweck, 1999).

While studies have not looked at curiosity, mindset, and achievement goals together, recent work found positive relations between their attitude towards epistemic curiosity (perceived value of inquisitive thinking), ability beliefs, and achievement goal orientations through a longitudinal intervention with Dutch fourth–sixth grade students (corresponding to ages ~7–10; Post & van der Molen, 2021). While their definition of curiosity focuses on perceived value, as opposed to trait curiosity, their findings reinforce the theoretical expectation that beliefs about ability and motivation are interlinked and malleable and highlight the value of testing these relationships.

The ages of 6 to 10 represent a critical developmental window when children begin formal schooling, encounter new academic and social challenges, and form foundational beliefs about learning and ability. Yet, much past research is limited to adult samples, and studies with younger children suggest developmental effects but currently show inconsistencies. Further, these constructs might be conceptualized differently by younger children and have different influences on their development, such as with achievement goal orientations. These might be linked to social comparisons in older children (Ruble et al., 1980), yet younger children demonstrate sensitivity to evaluative contexts and begin to adopt purposes for achievement that differentiate between learning and demonstrating competence (Butler, 1987). Social comparisons may also play a role in later mindset beliefs, but research shows very early development influences, such as parents’ language at age two predicting the development of mindset beliefs in eight-year-olds, yet children’s general mindset beliefs at this age are unclear. For example, findings on growth mindset are mixed: some studies indicate that younger children hold more fixed views of ability than older children (e.g., Pomerantz & Saxon, 2001; Stipek & Gralinski, 1996), while others find the opposite pattern or no developmental change at all (e.g., Kinlaw & Kurtz-Costes, 2007; Heyman & Giles, 2004). Similarly, work on curiosity has produced inconsistent age results—some studies report age-related declines in children’s curiosity (e.g., Engel, 2011; Engelhard & Monsaas, 1988), consistent with a developmental shift from the exploration to exploitation of knowledge (Liquin & Gopnik, 2022, whereas other research finds little or no evidence of change across development (e.g., J. Jirout & Klahr, 2012; Evans & Jirout, 2023).

Classroom practices that emphasize grades, correctness, or public evaluation—practices that become more common as standardized testing is introduced around the second grade (when children are about 8–9 years of age)—are suggested to be one explanation for the perception of the decline in curiosity across grades (J. J. Jirout et al., 2018) and have also been shown to increase performance-oriented patterns of affect and behavior even in young children (Ames, 1992; L. H. Anderman & Anderman, 1999). Research on achievement goals reveals both stability and change across development, with some mixed findings. For instance, studies show moderate to high stability in children’s mastery and performance goal orientations over time (e.g., L. H. Anderman & Anderman, 1999; Wigfield et al., 2007), suggesting that individual differences in goal orientation are relatively consistent. However, mean-level changes indicate that, on average, mastery goal orientation tends to decline from elementary school through to college (e.g., Meece & Miller, 2001; Bong, 2001; Fryer & Elliot, 2007), while findings on performance goal orientations are more mixed. Some studies report decreases (L. H. Anderman & Anderman, 1999; Bong, 2001) while others find little change (E. M. Anderman & Midgley, 1997). Taken together, these mixed findings underscore the need for more research on these constructs in early and middle childhood. Despite the mixed findings, children do hold beliefs about why they are learning in school and about whether their ability to learn is fixed or malleable, and there are individual differences in their likelihood of becoming curious, and thus understanding the developmental patterns of these constructs and their associations is important.

Studying these beliefs in tandem offers a richer account of how different methods may spark and sustain these dispositions in children’s learning. It is also important to understand the development of these constructs from the start of formal schooling, when children first experience and develop ideas about what school is about, and how these change as they become accustomed to the educational culture of classroom-based learning and academic assessments. For these reasons, the current study focuses on ages 6–10. In identifying shared methods for promoting these dispositions, future work can focus on combining efforts to support curiosity, growth mindset, and goal orientation. Yet, to understand these relations, a key first step is needed: ensuring each construct is distinct in younger children. Curiosity, growth mindset, and mastery goal orientation in particular share many underlying processes such that they may result in similar behaviors. For example, when a child eagerly engages in a difficult task, is it because they are curious, believe they can improve, or want to master it? The motivational expressions may look very similar and therefore warrant distinction. We contribute to the prior literature by exploring the following:

**RQ1.**  *Can curiosity, growth mindset, and achievement goals be measured as distinct constructs?*

**RQ2.**  *How do curiosity, growth mindset, and achievement goals relate to each other?*

**RQ3.**  *How does age relate to children’s curiosity, growth mindset, and achievement goal orientation?*

There is a limited availability of reliable tools for assessing these constructs in young children. As Evans et al. (2023) note, few validated self-report measures of curiosity exist for preschool and early elementary ages. Similarly, the existing mindset measures for this age group have notable psychometric and conceptual limitations (Muradoglu et al., 2024). Further, for mindset specifically, although often treated as a unitary construct, beliefs about ability can differ in meaningful ways—for example, in whether ability is seen as stable across time or responsive to environmental change. These distinctions are especially important in childhood, when children are actively constructing theories about how traits work. To capture this complexity, the measure we chose assessed two core dimensions of mindset: beliefs about the instability of ability and its malleability in response to environmental changes, such as changing to a new school that allowed them to build on their ability. Overall, we selected measures that best align with 6-to-10-year olds’ developmental level, and for growth mindset, using domain-specific items and collapsing across domains to reflect a general mindset construct. This age range corresponds to the early elementary school years, when children are transitioning into formal schooling, developing metacognitive skills that allow them to reflect on their own learning, and experiencing rapid growth in curiosity and motivation.

We hypothesized positive associations between curiosity and both growth mindset and mastery goal orientation (e.g., Hong et al., 2017; Grajcevci & Shala, 2021; Muradoglu et al., 2025). People with increased growth mindset orient towards learning goals such as increasing the mastery of skills (Blackwell et al., 2007; Elliott & Dweck, 1988); thus, we hypothesize positive associations between growth mindsets and mastery goals, but not performance goals (approach or avoid). Although there are some reasons to expect that curiosity declines with age (Engel, 2011), the evidence is inconsistent so we did not hold specific hypotheses about curiosity and age, though we did hypothesize that older children will have a stronger growth mindset (Muradoglu et al., 2024). Lastly, we hypothesize that as children grow older, they will orient more strongly towards performance goals (E. M. Anderman et al., 2002), with some stability in mastery goals (Middleton et al., 2004).

## 2. Materials and Methods

### 2.1. Participants

A total of 212 U.S. children participated (girls = 107, boys = 104, non-binary = 1; M_age_ = 8.31 years, SD = 1.37 years, range = 6.01 to 10.89; 57% White, 20% Asian, 14% Multiracial, 4% Black or African American, 4% identified with another race, and <1% American Indian or Alaska Native; <1% did not respond). In total, 78% of the sample identified as not Hispanic or Latino, 13% identified as Hispanic or Latino, and 9% did not respond. Our sample was highly educated with 90% of primary caregivers having a 4-year college degree or higher.

### 2.2. Procedure

All protocols were reviewed and approved by the Institutional Review Board. Children were recruited through social media and a participant database. Parental informed consent was collected via Qualtrics (Provo, UT, USA). Children completed the study via Zoom (San Jose, CA, USA), including curiosity, growth mindset, and achievement goal scales (added partway through data collection; N = 135).

### 2.3. Measures

**Figure 1:**
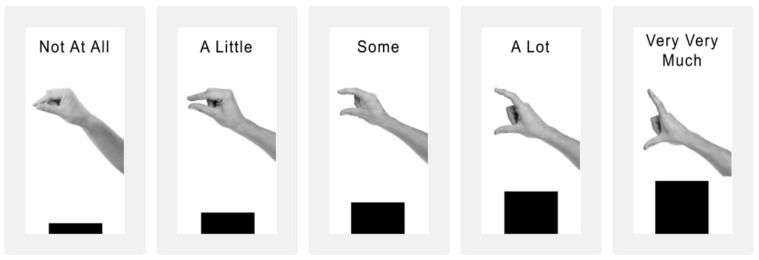
Graphical scale used for curiosity and achievement goal measures.

Measures used a Qualtrics-style survey showing items sequentially (37 total). Children completed the following measures in order, indicating their response to the researcher for each item (see Table 1). The questions were presented one at a time taking up the full screen shared via Zoom, and there was pre-recorded audio with initial instructions about how to use the graphical rating scale (Figure 1).

#### 2.3.1. Curiosity

Children heard a pre-recorded audio of 6 items (α = 0.773, McDonald’s ωs = 0.78) one at a time, assessing their general curiosity (Evans & Jirout, 2023) using a 5-point graphical scale asking how much each item is like them (e.g., “I ask questions to learn more about things”; see Figure 1). Mean scores were used, with higher values indicating higher curiosity.

#### 2.3.2. Growth Mindset

Children responded to 12 items (presented as slides with pictures and text read by a researcher) from a growth mindset scale (Muradoglu et al., 2024) assessing beliefs in math, drawing, and spelling. For each subject, the experimenter told the child a story about two characters who were not very good at that subject. Items measured instability (ability can change) using items such as “Will it always be this way? Will Jamie and Riley always be not very good at math?” followed by a confidence measure (i.e., “How sure are you?”). The story then explained the characters went to a new school where they practiced the subject a lot and measured malleability (ability can improve with effort) with items such as “Jamie and Riley were at this school for a long time. When they left this school, were they good at math or not good at math?” followed by a confidence measure (i.e., “Were they sort of [not] good, [not] good, or really [not] good”). Children received subjects/domains in a randomized order. Responses were scored using a 6-point rubric (Muradoglu et al., 2024), with higher scores reflecting stronger growth mindsets, averaged across domain items.

#### 2.3.3. Achievement Goals

The achievement goal scale is age-adapted from Patterns of Adaptive Learning Scales (Midgley et al., 2000) with three sub-scales (all Cronbach’s alpha and McDonald’s omega values > 0.78): mastery goal orientation (e.g., “One of my goals in class is to learn as much as I can.”; 5 items), performance approach (e.g., “One of my goals is to show others that I’m good at my class work.”; 5 items), and performance avoidance (e.g., “One of my goals is to keep others from thinking I’m not smart in class.”; 4 items). All items were read aloud, one at a time, by an experimenter and children were asked how much each item is like them on a 5-point graphical scale (see Figure 1), averaged for each sub-scale.

## 3. Results

All analyses were conducted in Stata 18.0 version 13 (College Station, TX, USA; https://www.stata.com/install-guide/windows/download/, accessed on 20 December 2025) and used listwise deletion, such that cases with missing data on any included variables were excluded.

### 3.1. RQ1: Can Curiosity, Growth Mindset, and Achievement Goals Be Measured as Distinct Constructs?

A series of Exploratory (EFA) and Confirmatory Factor Analyses (CFAs; see Appendix A) indicated that a 6-factor solution (curiosity, growth mindset—malleability of ability, growth mindset—instability of ability, mastery goal orientation, performance approach orientation, and performance avoidance orientation) provided the best fit to the data: χ^2^(284) = 414.44, *p* < 0.001; RMSEA = 0.06; TLI = 0.88, CFI = 0.90; SRMR = 0.07. All constructs demonstrated acceptable internal consistency (McDonald’s ωs > 0.77) and were statistically distinct. While fit indices were at or outside of commonly accepted cutoffs, research suggests that SEM fit indices are sensitive to different types of models and poor fit estimates tend to happen when residuals are correlated (Clark & Bowles, 2018; Fan & Sivo, 2007; Montoya & Edwards, 2021), which, with the conceptual overlap of constructs, is likely the case here. To further support the factor structure, we conducted Horn’s parallel analysis in Stata 18.0 version 13 (paran package, 1000 iterations, mean-based eigenvalues). The first six components had adjusted eigenvalues above 1 (4.86–1.09), meeting the standard retention criterion and confirming our initial six-factor solution based on the eigenvalue > 1 rule. All cross-loadings were below 0.4 other than performance avoidance, for which three of the four items cross loaded onto performance approach (attempts to combine factors did not improve model fit, see Appendix A.2). As in prior work (Muradoglu et al., 2024), growth mindset separated into two related but distinguishable facets: beliefs about the malleability of ability (ability can improve with effort) and beliefs about the instability of ability (ability can change over time). Therefore, for all following analyses, we separated growth mindset into two separate dimensions.

### 3.2. RQ2: How Do Children’s Curiosity, Growth Mindset, and Achievement Goal Orientation Relate?

See Table 2 for correlation values. Children’s curiosity positively related to both growth mindset instability beliefs (*p* = 0.009) and mastery goal orientation (*p* < 0.001), but not growth mindset malleability beliefs (*p* = 0.08), performance approach (*p* = 0.294), or avoidance (*p* = 0.736). All achievement goal sub-scales were positively correlated (*p*s < 0.001). Growth mindset and mastery goal orientation (*p* = 0.067), performance approach (*p* = 0.954), and performance avoidance (*p* = 0.176) were not significantly related.

### 3.3. RQ3: How Does Age Relate to Children’s Curiosity, Growth Mindset, and Achievement Goal Orientation?

Children’s growth mindset instability beliefs (*p* = 0.030) and performance approach (*p* = 0.012) increased with age, but curiosity (*p* = 0.945), growth mindset malleability beliefs (*p* = 0.304), mastery goal orientation (*p* = 0.159), and performance avoidance orientation (*p* = 0.064) were not significantly associated with age (see Table 2).

### 3.4. Exploratory RQ: Controlling for Age, Is Curiosity Predicted Separately by Both Growth Mindset and Mastery Achievement Goals?

Because curiosity was associated with both growth mindset instability beliefs and mastery orientation, and age was related to growth mindset, we tested the extent to which each of these variables related to curiosity scores when tested together using a regression analysis, controlling for age. The model was significant (F(3, 130) = 10.09, *p* < 0.001), explaining a meaningful portion of variance (*R*^2^ = 0.19). Only mastery goals significantly predicted curiosity (*p* < 0.001); growth mindset instability beliefs (*p* = 0.700) and age (*p* = 0.532) were not significant when included together. The lack of association with growth mindset instability beliefs and mastery goals indicates that these associations do not suggest the mediation of the mindset–curiosity association by mastery goals or other potential directional paths.

## 4. Discussion

Motivation is important in education and declines with schooling beginning early on, and young children’s motivational beliefs have long-term impacts on their later motivation and academic achievement. Thus, it is important to understand how different motivational constructs develop in children, whether they can be measured reliably in younger populations, and their associations in order to identify effective timepoints and methods of supporting or intervening to support children’s motivation. This study explores curiosity, growth mindset, and achievement goal orientations, which have all been studied independently or in pairs in past research but not yet together, and assessed these in a single sample of children younger than in past research. Thus, this work contributes not just new information about development and associations among these constructs but also tests whether they can be measured as distinct characteristics of students.

Curiosity, growth mindset (split into malleability and instability of ability), mastery, performance approach, and performance avoidance were confirmed as distinctly measured by both EFA and CFA, though performance goals had some cross-loadings, with avoidance factoring both with approach and as its own factor. It is possible that the cross-loadings are due to exploratory and confirmatory analyses being run on the same pool of participants due to the smaller sample size. Conceptually, the overlap between avoidance and approach orientations may also reflect children’s tendency to frame goals in socially comparative terms. Importantly, attempts to combine these factors did not improve fit and the six-factor solution, with performance approach and avoidance separated, remains consistent with the theory and prior work.

The distinction across constructs is important, as children’s outward behaviors may appear similar (e.g., engagement in a challenging task), but stem from fundamentally different motivational sources, even in elementary-age children. We did not assess academic outcomes, but other work has found each of these constructs to predict achievement (e.g., Shah et al., 2018; Claro et al., 2016; Nicholls, 1984), and it is important to know whether each contributes to distinct variability in motivation to inform future motivational support, as their associations were not all significant and not especially strong. As hypothesized, children who were more curious also had stronger growth mindset and mastery goal orientation. Performance approach and avoidance goal orientations were positively related, and both related to mastery goal orientation. Different goal types can co-occur, and these constructs may all tap into a more general continuum of being generally more or less motivated (e.g., Barron & Harackiewicz, 2001; Harackiewicz et al., 2002). Surprisingly, we did not observe relations between children’s growth mindset and their achievement goals, which contradicts past research and theory (e.g., Burnette et al., 2013; Dweck & Leggett, 1988; Elliott & Dweck, 1988; Muradoglu et al., 2025).

Curiosity is often assumed to relate to growth mindset (J. J. Jirout et al., 2018); curiosity is defined as an intrinsic motivation to learn, but this motivation likely requires the initial belief that one is capable of unlimited learning with effort, as indicated by holding growth mindset beliefs. However, evidence for this link is limited—only one study with sixth grade Chinese elite game players supports this link (Hong et al., 2017). Supporting the theorized associations between curiosity and mastery goals, prior evidence shows this link in adults (Litman, 2008). Our findings show that both mastery orientation and growth mindset instability—but not malleability—beliefs are related to curiosity in younger children. One possibility for this finding is that curiosity is more closely tied to an openness to change (captured by instability) than to beliefs about the specific mechanisms that produce change (captured by malleability). Malleability may require reasoning about specific causal mechanisms (e.g., practice, effort), which may feel more instructional than intrinsically motivating. For younger children especially, the idea that something might change could be more engaging than knowing why or how it would. Interestingly, only mastery goal orientation remained significantly related to curiosity when tested together with mindset beliefs. Mastery-oriented achievement goals and curiosity are aligned in that both motivate individuals to seek information for its intrinsic value rather than to achieve external outcomes. Perhaps growth mindset is a weaker influence on curiosity than mastery goal orientation because growth mindset beliefs function more distally, by framing learners’ general beliefs about the malleability of ability. Mindset beliefs influence how learners interpret challenges, but they do not themselves constitute a direct motivational mechanism for information seeking in the way that mastery goals and curiosity do. Future research should further explore potential mediation effects to better understand these associations, as these constructs are likely interrelated, as each relates to intrinsic motivation, positive emotional experiences during learning, and achievement in multiple ways (e.g., Ng, 2018; Grajcevci & Shala, 2021; Vogl et al., 2021). Further research can further explore contributions to learning and shared underlying processes or mechanisms to inform future intervention work.

Unexpectedly, growth mindset and mastery goal orientation did not relate, contradicting much prior research that has often found a positive association between these constructs (e.g., Burnette et al., 2013). This may stem from the fact that a growth mindset focuses on children’s beliefs about ability, whereas mastery goals relate to the reason for engaging in tasks, differing in the ways each are thought to influence motivation (Eccles & Wigfield, 2020). Much prior work assessed goals through behavior, such as goals measured by whether children choose to explore when there was more to be learned but more risk of failure or challenge (e.g., Elliott & Dweck, 1988; Smiley & Dweck, 1994), or mindset inferred by children’s persistence after challenge or failure (e.g., Diener & Dweck, 1980). The current study used self-report, as we were interested in children’s perceptions and views of themselves as learners and of learning contexts, which may not reflect their actual behaviors during learning tasks, and some research suggests the value of self-report measures for similar constructs (e.g., Boon-Falleur et al., 2022). More research is needed to understand how different types of beliefs and learning/mastery goals influence behavior (e.g., Sik et al., 2024) in actual learning contexts.

Although both belief types are likely influenced by similar factors, such as past experiences and attributions, they may also differ in their motivational roles. Consider, for instance, a high-ability math student who believes intelligence is fixed but still engages in tasks to demonstrate competence. For this student, a fixed belief may coexist with a competence-driven goal because their ability level allows them to succeed and develop competence. In contrast, a low-ability student who also believes intelligence is fixed may avoid competence-based goals altogether—if they believe they cannot improve, pursuing mastery may not seem worthwhile. This dynamic aligns with Expectancy-Value Theory, which posits that motivation is shaped by both the expectation of success and the value placed on the task (Eccles & Wigfield, 2020). A student’s perceived ability influences their expectancy for success, which in turn affects their goal orientation. Thus, ability level may moderate the relationship between mindset and goals, with fixed beliefs potentially supporting mastery goals only when students feel capable of achieving them. Another consideration is the potential importance of domain, as prior work suggests the domain-specificity of some motivational constructs (e.g., Peterson & Cohen, 2019; Limon, 2006), and the current measures included different domains for the mindset measure but not the other two. Similarly, because this pattern contrasts with established findings (e.g., Burnette et al., 2013; Dweck & Leggett, 1988; Muradoglu et al., 2025), careful consideration of measurement is warranted, especially construct validity. The absence of a correlation may reflect the possibility that younger participants interpreted the items in ways not fully aligned with the constructs’ intended operationalization. Understanding these dynamics through more research can provide insights into how children set and pursue their learning goals.

Age positively related to stronger growth mindset instability beliefs, but not curiosity nor growth mindset malleability beliefs. Although prior evidence is somewhat mixed, with some evidence that achievement goals and intrinsic motivation decline with age (Scherrer & Preckel, 2019), our growth mindset instability belief–age findings are consistent with prior research that older children are more likely to believe that ability and intelligence can change (Muradoglu et al., 2024). As children mature, their growing capacity for causal reasoning may support more nuanced beliefs about effort and environmental influence, leading to more of a growth mindset. The results here suggest that children might notice this change over time, represented by the instability belief change, but may not have ideas about how this change occurs, reflected in the lack of change in malleability beliefs. Further research about the reliability of this association with age and potential explanations for it are needed to explore what it could mean for the potential support of developing growth mindset beliefs.

Prior research on declining curiosity has focused on school-based contexts, whereas our general self-perception measure aligns with studies showing no age effect on curiosity (J. Jirout & Klahr, 2012). This pattern suggests that curiosity may be shaped more by context than by age, underscoring the need to create environments—especially in schools—that protect and promote children’s intrinsic interest as children progress through formal education. This is especially important considering that some studies suggest that children do not demonstrate curiosity in school contexts (e.g., Engel, 2011) especially compared to at home (Tizard & Hughes, 1984), and may not see curiosity as something that is even acceptable in school contexts (Post & van der Molen, 2018).

Contrary to our hypothesis, we found that older children orient less towards performance approach achievement goals, despite much of the literature showing that older children rely more on social comparison (Ruble et al., 1980). At the same time, some work has reported similar patterns to ours, with older children less likely to espouse performance goals (Kinlaw & Kurtz-Costes, 2007). One explanation is that they become more sensitive to social desirability (Millham & Jacobson, 1978), and may avoid endorsing items that emphasize outperforming others, even if such motives remain present. Another possibility is that they shift towards more internalized motivational frameworks, orienting towards their own standards for competence rather than comparison to others. Future work should explore these dynamics further with behavioral measures and examine how these constructs evolve longitudinally starting at a young age and across contexts.

The current sample (children of highly educated parents) limits the generalizability of the results. Parent education is often used as an indicator for socioeconomic status (SES) which has implications for student attitudes and beliefs (e.g., Claro et al., 2016). While some motivational processes may be consistent across SES groups (Yeager et al., 2019), baseline differences could influence how these constructs relate in different samples. Another limitation concerns our reliance on single-informant child reports, which may introduce method bias. In addition, correlation estimates based on self-report data can be noisy, so observed associations should be interpreted cautiously and replicated in larger, multi-method samples. Future work should replicate these associations with other populations, use behavioral tasks, assess domain-specific relations, examine longitudinal development, and explore additional motivational constructs and profiles (e.g., Yu & McLellan, 2020). Taken together, this work contributes to the current literature by measuring these constructs in younger children and added curiosity to the more researched constructs of mindset and achievement goal orientation, showing new correlational patterns to consider in future research. Though there are clear patterns of associations, inconsistencies in the prior research and our findings suggest the need for further work in this area. Critically, given the correlational nature of our research, we are not able to assume the directionality of these related constructs. Establishing directionality, such as whether growth mindset fosters curiosity or vice versa, would inform the design of targeted interventions. For example, if openness to change (instability) precedes curiosity, early interventions could focus on helping children recognize that ability is not fixed, knowing that it may also support curiosity. If mastery goals drive curiosity, then supporting competence may be a more effective entry point. Finally, it is important to understand how, individually and when interacting, each of these factors can support learning and more general academic enjoyment and success.

## Figures and Tables

**Table 1:** Curiosity, growth mindset, and achievement goal orientation measures. Parentheses after each item indicate exploratory factor analysis factor loadings.

**Curiosity** (Evans & Jirout, 2023)
*How much is this like you?* Not at all (1), A Little (2), Some (3), A Lot (4), Very Very Much (5)
I ask questions to learn more about things. (0.67) Curious kids like to figure out mysteries and learn new things and ask questions. How curious are you? (0.71) I am excited to learn new things. (0.73) When I don’t know something, it makes me want to learn more. (0.67) When something is surprising, I want to know more about it. (0.40) I enjoy discovering something new. (0.76)
**Growth Mindset** (Muradoglu et al., 2024) Modified to include two characters, one of each gender, to mitigate gender biases across domains. Example scale items for math subject. Other subjects include spelling & drawing.
**Instability of Ability**
*This is Jamie and Riley. And here’s something about Jamie and Riley: Jamie and Riley aren’t very good at math. Jamie and Riley get a lot of math problems wrong on their schoolwork. I just want to make sure you were paying attention: Are Jamie and Riley good at math? Or not good at math?* [corrective feedback provided] Now here’s a question for you: Will it always be this way? Will Jamie and Riley always be not very good at math? (Yes/No) How sure are you about this? Are you sort of sure? Or really sure? Factor Loadings: Math: 0.82, Spelling: 0.71, Drawing: 0.75
**Malleability of Ability**
*Now let me tell you what happened with Jamie and Riley. When Jamie and Riley were a little older, they moved to a school far away. At this school, kids do a lot of math. After Jamie and Riley started at this far-away school, they got to practice math a lot. Jamie and Riley did a lot of math at this school.* Now here’s a question for you: Jamie and Riley were at this school for a long time. When they left this school, were they good at math or not good at math? (Good/Not Good) Were they sort of [not] good, [not] good, or [not] really good? Factor Loadings: Math: 0.86, Spelling: 0.90, Drawing: 0.86
**Achievement Goal Orientation** (age adapted Midgley et al., 2000)
*How much is this like you?* Not at all (1), A Little (2), Some (3), A Lot (4), Very Very Much (5)
**Mastery Goal Orientation**
It’s important to me that I learn a lot of new things this year. (0.79) One of my goals in class is to learn as much as I can. (0.60) One of my goals is to get very good at a lot of new skills this year. (0.66) It’s important to me that I improve my skills this year. (0.68) It’s important to me that I understand my class work completely. (0.72)
**Performance Approach**
It’s important to me that other students in my class think I am good at my class work. (0.83) One of my goals is to show others that I’m good at my class work. (0.73) One of my goals is to show others that class work is easy for me. (0.78) One of my goals in school is to look as smart or smarter than other students in my class. (0.84) It’s important to me that I look smart compared to others in my class. (0.78)
**Performance Avoidance**
It’s important to me that my classmates do not think I am bad at my class work. (0.41) One of my goals is to keep others from thinking I’m not smart in class. (0.43) It’s important to me that my teacher doesn’t think that I know less than others in class. (0.65) One of my goals in class is to avoid looking like I have trouble doing the work. (0.74)

**Table 2:** Correlation table (*p*-values) for child curiosity, growth mindset sub-scales, and achievement goal orientation sub-scales. Values with * indicate *p*-values below 0.05.

Variables	N	M	(1)	(2)	(3)	(4)	(5)	(6)
(1) Age	212	8.31						
(2) Curiosity	210	3.63	−0.005					
			(0.945)					
(3) Growth Mindset: Instability	212	2.16	0.147 *	0.180 *				
			(0.030)	(0.009)				
(4) Growth Mindset: Malleability	212	2.25	0.070	0.121	0.636 *			
			(0.304)	(0.081)	(0.000)			
(5) Mastery Goal	135	3.93	−0.122	0.431 *	0.134	0.121		
			(0.159)	(0.000)	(0.121)	(0.163)		
(6) Performance Approach	135	2.79	−0.215 *	0.091	0.052	−0.041	0.327 *	
			(0.012)	(0.294)	(0.552)	(0.634)	(0.000)	
(7) Performance Avoid	135	2.74	−0.160	0.029	0.167	0.026	0.305 *	0.628 *
			(0.064)	(0.736)	(0.053)	(0.769)	(0.000)	(0.000)

**Figure A1:**
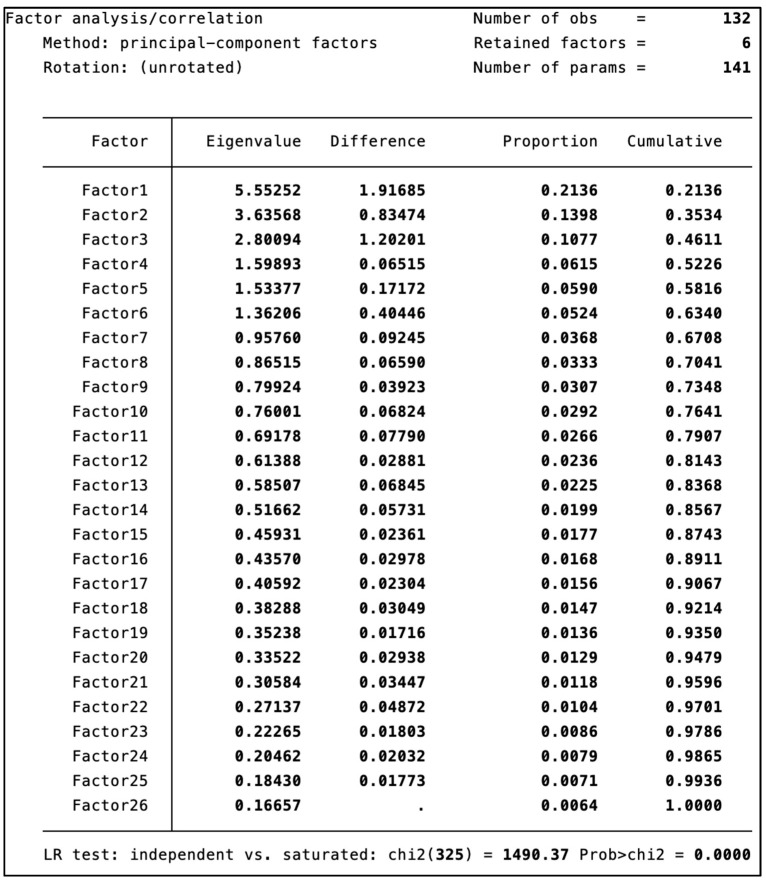
Principal component factors of exploratory factor analysis/correlation.

**Figure A2:**
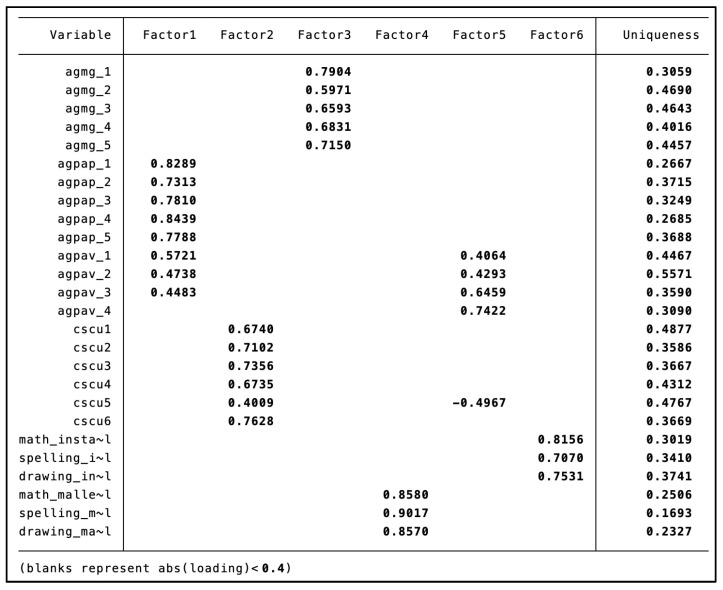
Rotated factor loadings and unique variances with loadings above 0.4 for exploratory factor analysis/correlation.

**Figure A3:**
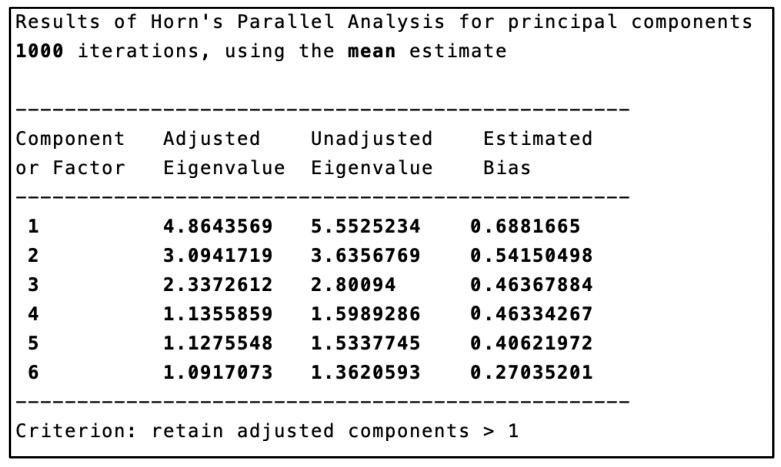
Results of Horn’s parallel analysis (paran package, Stata, 1000 iterations, mean-based eigenvalue estimation). Adjusted eigenvalues greater than 1 for the first six components supported the retention of a six-factor solution.

## Data Availability

The original data presented in the study are openly available in the Open Science Framework repository at https://osf.io/q386w/overview?view_only=487440192f094cda947b2d6a091bbe4e (accessed on 3 November 2025).

## Appendix A

### Appendix A.1

The exploratory factor analysis correlation and factor loadings were produced in Stata (see Figure A1 and Figure A2).

### Appendix A.2

The confirmatory factor analysis goodness of fit statistics were produced in Stata (see Table A1).

**Table A1:** Confirmatory factor analysis goodness of fit statistics for uni-, five- [mindset combined and performance combined models], and six-factor models.

Model	Goodness of Fit Indices
Uni-Factor: One singular construct	
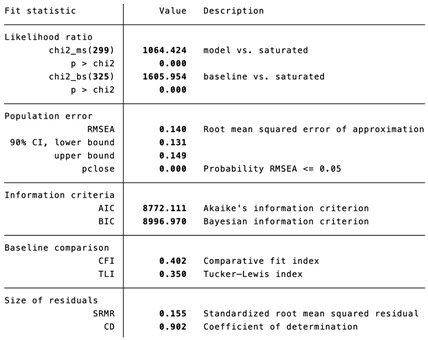
Five-Factor Growth Mindset Combined: Curiosity, Growth Mindset, Mastery Goal Orientation, Performance Approach Orientation, and Performance Avoidance Orientation	
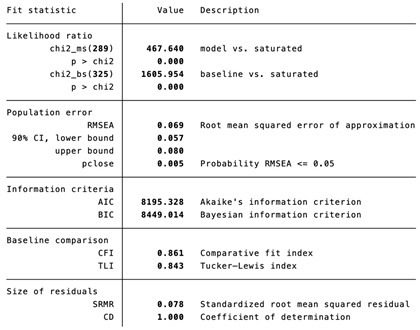
Six-Factor: Curiosity, Growth Mindset—malleability of ability, Growth Mindset—instability of ability, Mastery Goal Orientation, Performance Approach Orientation, and Performance Avoidance Orientation	
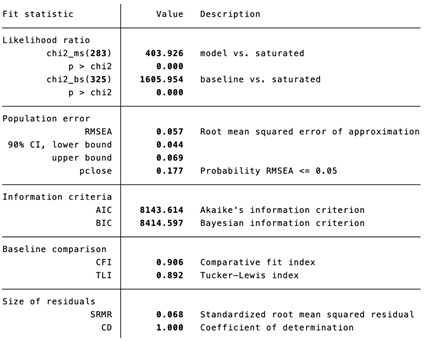
Five-Factor Performance Combined: Curiosity, Growth Mindset—malleability of ability, Growth Mindset—instability of ability, Mastery Goal Orientation, Performance Orientation	
